# Chemogenetic inhibition of Grpr- and/or Npff-expressing spinal neurons in mice suppresses chloroquine-evoked itch but not signs of mechanical allodynia in a neuropathic pain model

**DOI:** 10.1097/j.pain.0000000000004024

**Published:** 2026-06-02

**Authors:** Erika Polgár, Maria Gutierrez-Mecinas, Allen C. Dickie, Mai Abu Hajer, Andrew H. Cooper, Greg A. Weir, Andrew J. Todd

**Affiliations:** a Spinal Cord Group, School of Psychology and Neuroscience, University of Glasgow, Glasgow, United Kingdom; b Department of Anatomy and Histology, School of Medicine, The University of Jordan, Amman, Jordan

**Keywords:** Spinal cord, Vertical cell, Excitatory interneuron, Spared nerve injury, Mechanical allodynia, Place preference assay

## Abstract

Mechanical hypersensitivity following peripheral nerve injury is common and difficult to treat. It has been suggested that this results from activation of a normally silent spinal cord circuit that links low-threshold mechanoreceptive primary afferents to nociceptive projection neurons in lamina I and that this pathway involves a class of excitatory interneurons known as vertical cells. We have recently identified 2 neurochemical types of vertical cells, based on expression of the gastrin-releasing peptide receptor (GRPR) and neuropeptide FF (NPFF). Although little is known about the role of NPFF cells, those that express GRPR are strongly implicated in itch; for example, it has been reported that ablation of GRPR cells suppresses itch but has no effect on pain. In this study, we have used chemogenetics to inhibit each population separately (in GRPR^CreERT2^ and NPFF^Cre^ mice) and both together (in GRPR^CreERT2^; NPFF^Cre^ mice). We found that the itch evoked by intradermal injection of chloroquine in the calf was suppressed in each case, but there was no effect on baseline mechanical sensitivity. In addition, we saw no effect of the chemogenetic inhibition on either mechanical hypersensitivity or on a measure of spontaneous pain in the spared nerve injury model of neuropathic pain. These results indicate that both of these populations of vertical cells are involved in the itch evoked by chloroquine but do not support a role for these cells in neuropathic mechanical hypersensitivity.

## Abstract 2

This study provides evidence that 2 molecularly defined vertical cell populations are required for itch, but not for neuropathic pain.

## 1. Introduction

Neuropathic pain is a potentially debilitating condition affecting ∼7% of the population^57,62^ and representing a major clinical burden. Symptoms include spontaneous pain, exaggerated pain (hyperalgesia), and pain evoked by normally innocuous stimuli (allodynia). Despite intensive research, the mechanisms underlying neuropathic pain remain poorly understood. Although aberrant peripheral input is a major driver of neuropathic pain,^23^ pathological changes in the central nervous system make an important contribution. For example, it has been proposed that mechanical allodynia involves low-threshold mechanoreceptive primary afferents gaining access to spinal cord circuits that normally convey information perceived as painful.^26,33,40,55^

Primary afferents arborise in a highly organised way within the spinal cord.^1,44,52,56^ Most fine diameter (C and Aδ) afferents function as nociceptors or thermoreceptors, and these terminate mainly in the superficial dorsal horn (SDH; laminae I-II). In contrast, larger diameter (Aβ) low-threshold mechanoreceptive cutaneous afferents (A-LTMRs) arborise in laminae III-V. Information carried by these afferents is transmitted through complex circuits involving dorsal horn interneurons and is conveyed to the brain by projection cells belonging to the anterolateral system (ALS).^5,52^ Anterolateral system neurons are concentrated in lamina I and are also present in the lateral spinal nucleus, the deep dorsal horn (laminae III-VI), and around the central canal.^14,58,59^ Ablation of neurons expressing the neurokinin 1 receptor (NK1r) in rats greatly reduced mechanical hypersensitivity in a neuropathic model.^36^ As NK1r is expressed by most lamina I projection neurons,^6,15,28,32,48,54^ this finding implicated lamina I ALS cells in mechanical allodynia. Further support for this suggestion came from the report that after peripheral nerve injury, many lamina I projection neurons showed novel responses to low-threshold mechanical stimuli.^25^

The great majority of neurons in SDH are interneurons, and ∼75% of these are excitatory.^43^ Superficial dorsal horn excitatory interneurons have been assigned to various populations, based on anatomical, electrophysiological, and molecular features.^13,20,22,35,42,45,53^ Grudt and Perl^20^ identified 3 main types, vertical, radial, and transient central cells, and we have shown that these correspond to classes defined by molecular properties.^13,35,39,42,45^ Vertical cells, located in lamina I and the outer part of lamina II (IIo),^20,39,42,45^ have attracted particular attention, as they have ventrally-directed dendrites that penetrate the A-LTMR-receiving zone,^1^ and axons that innervate lamina I projection neurons.^11,27^ It has been proposed that they connect low-threshold afferents to nociceptive lamina I ALS cells through a polysynaptic circuit that is normally closed, and that opening of this circuit underlies mechanical hyperalgesia/allodynia in neuropathic pain.^3,16,26,41^ We recently identified 2 partially overlapping neurochemical populations of vertical cells, based on expression of gastrin-releasing peptide receptor (GRPR) and neuropeptide FF (NPFF).^42,45^ Little is known about the function of NPFF-expressing neurons, although it was recently reported that chemogenetically inhibiting them did not reduce signs of neuropathic allodynia.^35^ Gastrin-releasing peptide receptor–expressing cells have been implicated in pruritogen-evoked itch.^34,38,50,51^ However, we found that chemogenetically activating GRPR cells resulted in behaviours that resembled both itch and mechanical allodynia,^42^ suggesting that they might be involved in both itch and pain mechanisms. In this study, we have used chemogenetic inhibition to investigate the roles of GRPR- and NPFF-expressing cells in pruritogen-evoked itch and neuropathic pain.

## 2. Methods

Experiments were approved by the Ethical Review Process Applications Panel of the University of Glasgow and were performed in accordance with the UK Animals (Scientific Procedures) Act 1986. All surgical procedures and behavioural tests were performed by individuals blinded to the genotypes of the mice. In behavioural tests and their analysis, experimenters were also blind to the treatment type (drug or vehicle).

### 2.1. Animals

We used a GRPR^CreERT2^ mouse line in which a sequence coding for a fusion protein consisting of codon-improved Cre recombinase (iCre) and mutated estrogen receptor was inserted into the GRPR locus^34^ and an NPFF^Cre^ line in which the sequences for the T2A peptide and the open reading frame of iCre were inserted between the last amino acid and the translation termination codon in exon 3 of the NPFF gene.^45^ Both of these Cre lines were on a C57BL/6J background. We generated 4 different genotypes: (1) GRPR^CreERT2^, (2) NPFF^Cre^, (3) GRPR^CreERT2^; NPFF^Cre^, and (4) wild-type (C57BL/6J). Because the GRPR gene is located on the X chromosome, all experiments involving GRPR^CreERT2^ mice involved either male mice that were hemizygous or female mice that were homozygous for the GRPR^CreERT2^ allele.

### 2.2. Intraspinal injections

**Figure 1.**
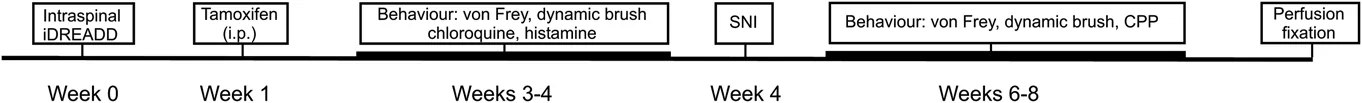
Experimental timeline. Mice belonging to each of the 4 genotypes (wild-type, GRPR^CreERT2^, NPFF^Cre^, and GRPR^CreERT2^; NPFF^Cre^, 7-9 mice per genotype) received intraspinal injections of AAV8.hSyn.Cre^ON^.hM4Di.mCherry (iDREADD) (week 0) and then received i.p. injections of tamoxifen in the following week. They then underwent initial behavioural testing (von Frey, dynamic brush, pruritogens). Spared nerve injury surgery (SNI) was performed in week 4, followed by further behavioural tests: von Frey, dynamic brush, and conditioned place preference (CPP). Following completion of behavioural tests, they were perfused with fixative under terminal general anaesthesia. Note that, in addition, a set of wild-type mice underwent SNI surgery and were tested for CPP with gabapentin, to provide a control for the CPP method.

The effects of chemogenetic inhibition on behaviour were tested on between 7 and 9 mice (including at least 3 of each sex) from each of the 4 genotypes listed above (Fig. 1). Mice were aged between 32 and 62 days at the start of the experiment. All of these mice received intraspinal injections of AAV coding for a Cre-dependent form of the inhibitory DREADD receptor (AAV8.hSyn.Cre^ON^.hM4Di.mCherry; Addgene #44362-AAV8) that were targeted to the L3, L4, and L5 segments on the right-hand side. The method for intraspinal injection was as described previously.^29,42^ Mice were anaesthetised with isoflurane (1%-2%) and placed in a stereotaxic frame. After surgical exposure, the bodies of T12 and L1 vertebrae were clamped. The L3 and L5 segments were accessed through the intervertebral spaces on either side of the T13 vertebra, while the injection into the L4 segment was made through a small hole drilled in the lamina of T13.^24^ Injections were made using glass micropipettes (outer tip diameter ∼60 µm) attached to a 10 µL Hamilton syringe, at 400 µm lateral to the midline and a depth of 300 µm below the spinal cord surface. At each site, a volume of 300 nL containing 2.2 × 10^8^ gene copies of virus was injected at a flow rate of 30 nL per minute, and after each injection the pipette was left in place for 5 minutes to minimise leakage back up the track. All of the mice received perioperative analgesia (buprenorphine 0.1 mg/kg and carprofen 10 mg/kg s.c.). In the week following intraspinal injections, all mice received tamoxifen (between 5 and 9 mg, split over 3 nonconsecutive days, i.p.).

### 2.3. Initial behavioural testing

The mice underwent an initial series of behavioural tests, which took place in the third and fourth weeks after intraspinal injection (Fig. 1). All mice had undergone handling and acclimatisation to the chambers and tests before the start of testing, and all tests of a particular type were carried out by the same experimenter. Mechanical sensitivity was assessed with a set of von Frey filaments, as described previously,^42^ and the 50% withdrawal threshold was determined by the up-down method.^7^ We also stroked the plantar surface of the hindpaw with a soft paintbrush (dynamic brush test^16^) and assigned scores as follows: 1 for sustained lifting of the paw, 2 for lifting of the paw above the level of the body, and 3 for flinching or licking of the paw. These tests were used both to assess the response of uninjured animals to clozapine-N-oxide (CNO)-mediated inhibition of GRPR and/or NPFF cells and to determine whether this response changed in the mechanically hypersensitive state that follows spared nerve injury (SNI; see below).

We tested itch behaviours in response to chloroquine and histamine injected into the right calf on separate occasions.^4,24^ For these pruritogen-evoked itch tests, mice were acclimatised for 30 minutes in plastic enclosures (10 × 10 × 10 cm, with an opaque divider between them) surrounded by angled mirrors to provide unobstructed views of the right hindlimb, and video recorded for a further 30 minutes. They then received intradermal injections consisting of 10 µL of 1% chloroquine or 1% histamine dissolved in phosphate-buffered saline (PBS) into the right calf, which had been shaved ∼24 hours before testing. Successful intradermal injection was confirmed by the appearance of a skin bleb at the injection site. Mice were video recorded for 30 minutes following pruritogen injection. Injections of chloroquine and histamine into an individual mouse were separated by at least 2 days, and in all cases, the chloroquine injection was performed before the histamine injection. Each of the behavioural tests described above was performed twice in consecutive weeks, once in the presence of CNO (5 mg/kg) and once in the presence of vehicle. In the pruritogen tests, the time spent biting the injected area was scored offline using BORIS event-logging software (freely available, https://www.boris.unito.it/),^18^ with videos viewed at one-fifth speed for analysis.^4^

A crossover design was used for all experiments in which CNO or vehicle was administered to test the effects of chemogenetic inhibition, and this was also the case for behavioural testing after spared nerve injury (see below). For each animal, the order of treatment with CNO or vehicle was randomised.

### 2.4. Spared nerve injury and associated behavioural testing

After completion of the tests described above, SNI surgery^12^ was performed on the mice (Fig. 1). They were anaesthetised with 1% to 2% isoflurane, and an incision was made in the skin over the right thigh. The tibial and common peroneal nerves on the right-hand side were identified, and 7-0 Mersilk (Ethicon) was used to apply a tight ligature to these nerves, with care taken to avoid manipulating or damaging the sural branch of the sciatic nerve. A length of nerve (approximately 2-3 mm) distal to the ligature was removed, and the wound was closed in layers. Again, all of the mice received perioperative analgesia (buprenorphine 0.05 mg/kg and carprofen 5 mg/kg s.c.). Approximately 2 weeks later, further behavioural testing with von Frey filaments and dynamic brush^16^ was conducted to assess mechanical hypersensitivity. In this case, the von Frey filaments were applied to the lateral side of the paw (sural nerve territory). Again, tests were performed twice, once following treatment with CNO and once following treatment with vehicle. For each animal, the same order of treatments (vehicle or CNO) was used as in the initial behavioural tests.

In addition, to assess ongoing neuropathic pain, we used a conditioned place preference (CPP) test with a biased chamber-assignment conditioning protocol, as described previously.^4,9^ The custom-built CPP testing apparatus consisted of 2 side chambers (170 × 150 mm) connected by a centre chamber (70 × 75 mm), 180 mm tall and with infrared-transparent plastic lids. Mice were able to discriminate between the 2 side chambers based on visual (vertical or horizontal black-and-white stripes on the walls) and somatosensory (rough or smooth-textured floors) cues. Mice were tested in groups of 6. In each case, the group consisted of 4 of the mice (GRPR^CreERT2^, NPFF^Cre^, GRPR^CreERT2^; NPFF^Cre^, or wild-type) that had received intraspinal injections of AAV8.hSyn.Cre^ON^.hM4Di.mCherry and undergone SNI, and these were tested for the effect of CNO on place preference. In addition, each group included 2 wild-type C57BL/6J that had undergone SNI (with the surgical procedure described above), and these were tested to assess the effect of gabapentin on CPP, in order to provide a positive control.^4^

On day 1 of the CPP testing (acclimation), mice had free access to explore all chambers for 45 minutes. On days 2 and 3 (preconditioning), mice were again allowed to freely explore for 45 minutes, and their positions were recorded using an infrared camera and AnyMaze 7.16 software (Stoelting). Mice spending more than 90% or less than 5% of time in either side chamber during preconditioning would have been excluded,^4^ but this did not apply to any of the mice in this study. For conditioning (days 4-6), mice received i.p. vehicle injection, were returned to their home cage for 5 minutes, and then were confined to their preferred side chamber for 45 minutes. Four hours later, mice received i.p. CNO (5 mg/kg, for those mice that had received intraspinal injections of AAV8.hSyn.Cre^ON^.hM4Di.mCherry) or gabapentin (100 mg/kg, for wild-type mice that had not received intraspinal injections). They were returned to their home cage for 5 minutes and were then placed in their nonpreferred chamber for 45 minutes. On the test day (day 7), mice could freely explore all chambers while their positions were recorded for 45 minutes, as during preconditioning. Difference scores were calculated by subtracting the mean preconditioning time from the time spent in each chamber on the test day.

### 2.5. Histology

Following completion of behavioural testing, the mice that had received intraspinal injections were deeply anaesthetised (20 mg pentobarbitone, i.p.) and fixed by intracardiac perfusion with 4% freshly depolymerised formaldehyde in phosphate buffer. Sagittal sections through the L3-5 segments were cut with a vibrating blade microtome and reacted with chicken anti-mCherry (1:10,000, Abcam, Cat# Ab205402) and guinea pig anti-NeuN (1:1000, Synaptic Systems, Cat# 266004), followed by donkey anti-chicken IgY conjugated to AlexaFluor 488 (1:500, Jackson Immunoresearch, Cat# 703-545-155) and donkey anti-guinea pig IgG conjugated to Rhodamine Red-X (1:200, Jackson Immunoresearch, Cat# 706-296-148). All antibodies were diluted in PBS containing 0.3 M NaCl, 0.3% Triton-X100, and 5% normal donkey serum. Sections were examined with a fluorescence microscope (Zeiss Axioscope 5) to confirm the extent of labelling with the mCherry antibody. Representative examples of injection sites were scanned with a Zeiss LSM710 confocal microscope equipped with argon multiline, 405-nm diode, 561-nm solid-state, and 633-nm HeNe lasers.

### 2.6. Exclusions from behavioural testing or analysis

Based on the histological findings, most behavioural results (von Frey tests, dynamic brush, and all tests conducted after SNI) were excluded from the analysis of mice in which any of the L3, L4, or L5 injection sites were not successful. However, if the L3 injection was successful but the L4 and/or L5 injections were not, the analysis of response to pruritogens was included, as these tests involved the L3 dermatome.

One GRPR^CreERT2^; NPFF^Cre^ mouse developed a mild, transient skin lesion on the ipsilateral calf to the spinal injection, which resolved completely by the time of the SNI operation. In this case, responses to pruritogen injection were not tested, but all other behavioural tests were performed and included in the analysis.

Due to restrictions on the availability of the CPP equipment, not all mice underwent CPP testing (Table 1). Testing was performed on 6 of the GRPR^CreERT2^, 6 of the NPFF^Cre^, 7 of the GRPR^CreERT2^; NPFF^Cre^, and 6 of the wild-type mice that had received intraspinal injections of AAV8.hSyn.Cre^ON^.hM4Di.mCherry and had undergone SNI. All of these mice were tested for the effects of CNO. In addition, CPP testing was carried out on 14 wild-type mice that had received SNI but no intraspinal injection, and these were tested for the effects of gabapentin.

**Table 1:** Numbers of animals of each genotype used in the study.

	Reflex tests			CPP		
	Total	Male	Female	Total	Male	Female
GRPR^CreERT2^	9	5	4*	6	4	2
NPFF^Cre^	9	4†	5	6	2	4
GRPR^CreERT2^; NPFF^Cre^	8	4	4	7	3	4
Wild type	7	3	4	6	3	3
Wild type (SNI only)				14	8	6

Rows 3 to 6 show the numbers of mice of each genotype that received intraspinal injections of AAV.Cre^ON^.hM4Di-mCherry, as well as the number that were tested with conditioned place preference (CPP) using CNO. Row 7 shows the number of wild-type mice that underwent SNI and were tested with gabapentin for conditioned place preference.

* In one (female) GRPR^CreERT2^ mouse, the L3 injection site was not labelled with mCherry, and this mouse was excluded from all behavioural analyses.

† In one (male) NPFF^Cre^ mouse, only the L3 injection was successful, and for this mouse, only behavioural data related to pruritogen tests were used.

### 2.7. Electrophysiology

To assess the action of CNO on GRPR- or NPFF-positive dorsal horn neurons that expressed hM4Di, electrophysiological recordings were performed on spinal cord slices that were obtained from 6 GRPR^CreERT2^ (3 female, 3 male) and 3 NPFF^Cre^ (2 female, 1 male) mice that had received bilateral injections of AAV8.hSyn.Cre^ON^.hM4Di.mCherry, targeted to the L3 segment. The GRPR^CreERT2^ mice received tamoxifen, as described above. At least 2 weeks following intraspinal injections, or in the case of GRPR^CreERT2^ mice, at least 2 weeks after the final dose of tamoxifen, mice were deeply anaesthetised with pentobarbitone (20 mg i.p.), perfused with ice-cold dissection solution, and the lumbar region of the spinal cord was isolated. This was embedded in low-melting-point agar (∼3%; Thermo Fisher Scientific, Paisley, UK), and parasagittal (300 μm) slices were cut with a vibrating-blade microtome (7000smz-2; Campden Instruments, Loughborough, UK). Immediately after cutting, the slices were incubated in an *N*-methyl-D-glucamine (NMDG)-based recovery solution at 32°C for ∼15 minutes and were then placed in a modified recording solution at room temperature for an additional 1 hour before being transferred to the recording chamber, where they were continually perfused with recording solution at a rate of ∼2 mL/minute. The solutions contained the following (in mM): Dissection, 3.0 KCl, 1.2 NaH_2_PO_4_, 0.5 CaCl_2_, 7.0 MgCl_2_, 26.0 NaHCO_3_, 15.0 glucose, 251.6 sucrose; NMDG recovery, 93.0 NMDG, 2.5 KCl, 1.2 NaH_2_PO_4_, 0.5 CaCl_2_, 10.0 MgSO_4_, 30.0 NaHCO_3_, 25.0 glucose, 5.0 Na-ascorbate, 2.0 thiourea, 3.0 Na-pyruvate, 20.0 HEPES; Modified recording, 92.0 NaCl, 2.5 KCl, 1.2 NaH_2_PO_4_, 2.0 CaCl_2_, 2.0 MgSO_4_, 30.0 NaHCO_3_,25 glucose, 5.0 Na-ascorbate, 2.0 thiourea, 3.0 Na-pyruvate, 20.0 HEPES; Recording, 125.8 NaCl, 3.0 KCl, 1.2 NaH_2_PO_4_, 2.4 CaCl_2_, 1.3 MgCl_2_, 26.0 NaHCO_3_,15 glucose. All solutions were bubbled with 95% O_2_/5% CO_2_.

Neurons were visualised using a fixed stage upright microscope (BX51; Olympus, Southend-on-Sea, UK) equipped with a 40× water immersion objective, infrared differential interference contrast (IR-DiC) illumination, and a CMOS camera (Prime BSI; Teledyne Photometrics, Birmingham, UK). Fluorescently labelled cells were visualised using a 550 nm LED (pE-100; CoolLED, Andover, UK) and targeted whole-cell patch-clamp recordings were made from mCherry-positive cells in the superficial dorsal horn. Patch-clamp pipettes were pulled from borosilicate glass (Warner Instruments, Holliston, MA) on a Sutter P1000 puller (Sutter Instruments, Novato, CA). These had a typical resistance of 4 to 8 MΩ when filled with an intracellular solution containing (in mM): 130.0 K-gluconate, 10.0 KCl, 2.0 MgCl_2_, 10.0 HEPES, 0.5 EGTA, 2.0 ATP-Na, 0.5 GTP-Na, and 0.2% Neurobiotin, pH adjusted to 7.3 with 1.0 M KOH. Data were recorded and acquired with a Multiclamp 700B amplifier and pClamp 10 software (both Molecular Devices, Wokingham, UK), and were filtered at 4 kHz and digitised at 10 kHz.

After achieving a stable whole-cell configuration, cells were voltage-clamped at −70 mV, and a series of 100 ms voltage steps from −70 to −50 mV (2.5 mV increments) was applied to determine the current-voltage relationship, which was used to calculate the resting membrane potential. Cells that had a resting membrane potential that was greater than −35 mV were excluded from all analysis. A series of five −5 mV steps (1-second duration), from a holding potential of −70 mV, was used to calculate input resistance. Rheobase was determined by applying a series of 1-second depolarising current steps of increasing amplitude (5 pA increments) in current-clamp mode. To assess the effect of hM4Di activation, CNO (25 µM) was bath-applied during an 8-minute gap-free current-clamp recording, comprising a 1-minute baseline followed by application of CNO for the remaining 7 minutes. Clozapine-N-oxide was continued while input resistance and rheobase were determined, as described above.

All chemicals were obtained from Sigma, except sucrose, glucose, NaH_2_PO_4_ (Thermo Fisher Scientific, Paisley, UK), NaCl, KCl, HEPES (VWR, Lutterworth, UK), CNO (Biotechne, Abingdon, UK) and Neurobiotin (Vector Laboratories, Peterborough, UK).

### 2.8. Statistics

Data are reported as mean ± standard deviation. Statistical analyses were performed using Prism V10 software (GraphPad Software, CA). Analysis of von Frey and dynamic brush behavioural tests was performed using a 3-way ANOVA with Tukey multiple comparison tests. Data from chloroquine and histamine itch tests were analysed using a 2-way ANOVA with Tukey multiple comparison tests. Analysis of CPP testing of spontaneous neuropathic pain was performed using a 2-way ANOVA (to determine the effect of CNO) or a paired *t* test (to determine the effect of gabapentin). Analysis of data obtained from electrophysiological recordings to study the effect of CNO on hM4Di expressing neurons was performed using a 2-way ANOVA with Šídák multiple comparisons tests. A *P* value of <0.05 was considered statistically significant, and significance is denoted within figures as follows: *P < 0.05, **P < 0.01, ***P < 0.001.

Required group sizes for von Frey and brush experiments were estimated using a power analysis (G*Power 3.1.9.7) based on data from prior experiments measuring mechanical hypersensitivity after SNI.^10^ To detect a hypothetical 33% increase in withdrawal thresholds after CNO administration with α = 0.05, power = 0.8, an effect size f = 0.316, 4 repeated measures (timepoints), repeated-measures correlation = 0.55, and Greenhouse-Geisser epsilon = 0.58, a group size of 8 was required. Von Frey testing was used to determine sample size, as it yielded a larger estimated group size than brush testing. Power analysis for CPP testing was based on prior experiments using gabapentin to induce CPP in neuropathic mice.^4^ To detect a Chamber × Group interaction with α = 0.05, power = 0.8, and an effect size *f* = 0.39, a group size of 6 was required.

## 3. Results

### 3.1. Injection sites

**Figure 2.**
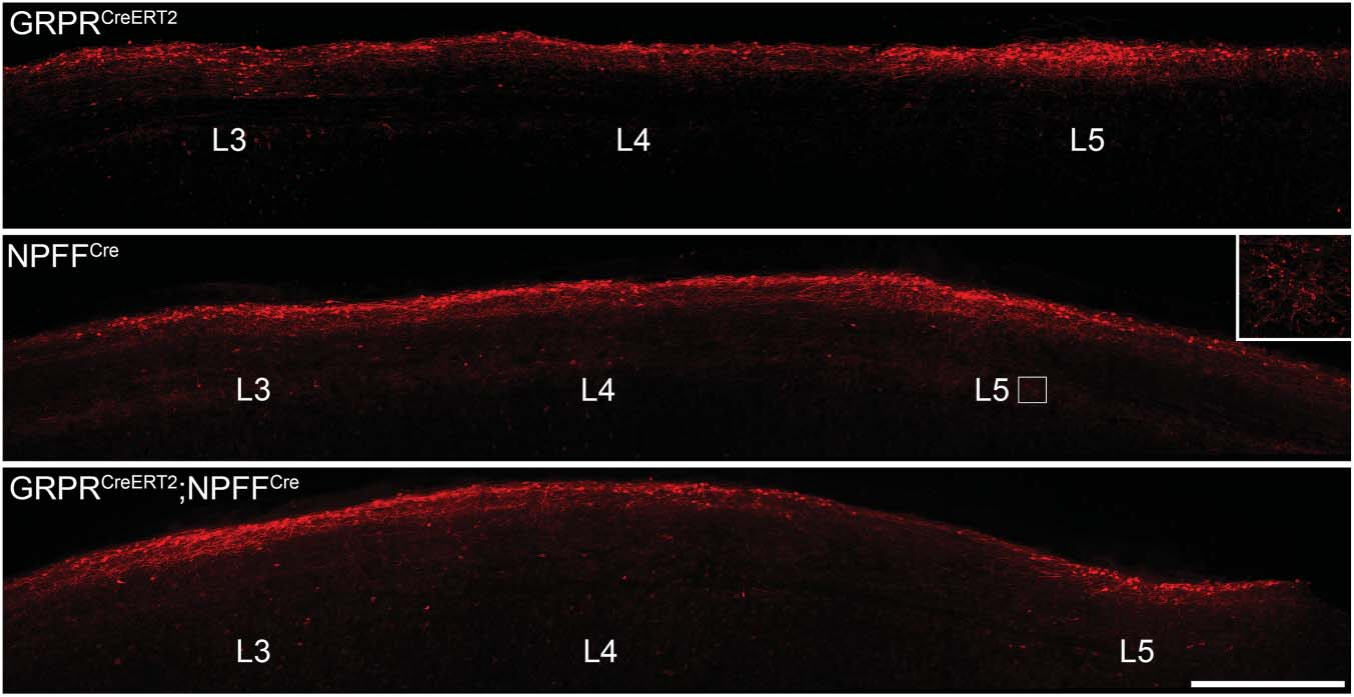
Examples of intraspinal injection sites. The images show sagittal sections through the lumbar enlargement of representative GRPR^CreERT2^, NPFF^Cre^, and GRPR^CreERT2^; NPFF^Cre^ mice that had been injected with AAV8.hSyn.Cre^ON^.hM4Di.mCherry. Sections were immunostained to reveal mCherry. Approximate locations of the injection sites targeted on the L3, L4, and L5 segments are indicated in each case. There is dense labelling in the superficial laminae, and in some cases, this forms an uninterrupted band extending across the 3 segments, while in others, there are “gaps” that contain relatively few labelled cells. A few labelled cells are present in deeper laminae. In all cases, mCherry labelling of axons and boutons was visible; an example is shown in the inset for the NPFF^Cre^ mouse, which is a higher magnification view of the area outlined by the box. Scale bar = 500 µm.

The total numbers of experiments involving the different genotypes are shown in Table 1. Overall, 9 GRPR^CreERT2^, 9 NPFF^Cre^, 8 GRPR^CreERT2^; NPFF^Cre^, and 7 wild-type mice received injections of AAV.Cre^ON^.hM4Di-mCherry targeted on the L3, L4, and L5 segments on the right side. Examination of the injection sites at the end of the behavioural testing revealed that in all but 2 cases among the GRPR^CreERT2^, NPFF^Cre^, and GRPR^CreERT2^; NPFF^Cre^ mice, all 3 injection sites contained numerous cells that were labelled with mCherry, indicating successful injections into each segment (Fig. 2). In 1 NPFF^Cre^ mouse, only the L3 injection was successful, while in 1 GRPR^CreERT2^ mouse only, the L4 and L5 injections were successful. The NPFF^Cre^ mouse with only the L3 segment injected was therefore included in the analysis for effects of chemogenetic inhibition on pruritogen-evoked itch, while the GRPR^CreERT2^ mouse with only L4 and L5 injections was excluded from all behavioural analyses. Although we were not able to assess injection sites in the wild-type mice (due to lack of Cre recombinase), the high success rate among Cre-expressing mice (24/26 successfully injected in all 3 segments) makes it likely that the great majority of these would also have been injected successfully. Immunostaining for mCherry revealed that labelled cells were mainly present in the superficial laminae, with scattered cells in deep dorsal horn. This was similar to the pattern observed when AAVs coding for other Cre-dependent constructs were injected into GRPR^CreERT2^ or NPFF^Cre^ mice.^42,45^ In many cases the band of mCherry labelling extended without interruption across the L3-L5 segments, while in others there were gaps between the injection sites that contained relatively few labelled cells (Fig. 2). In all genotypes, numerous mCherry-labelled axons were visible, and these could be recognised as elongated thin profiles with varicosities, corresponding to axonal boutons (Fig. 2).

### 3.2. The effects of clozapine-N-oxide on pruritogen-evoked itch behaviour

**Figure 3.**
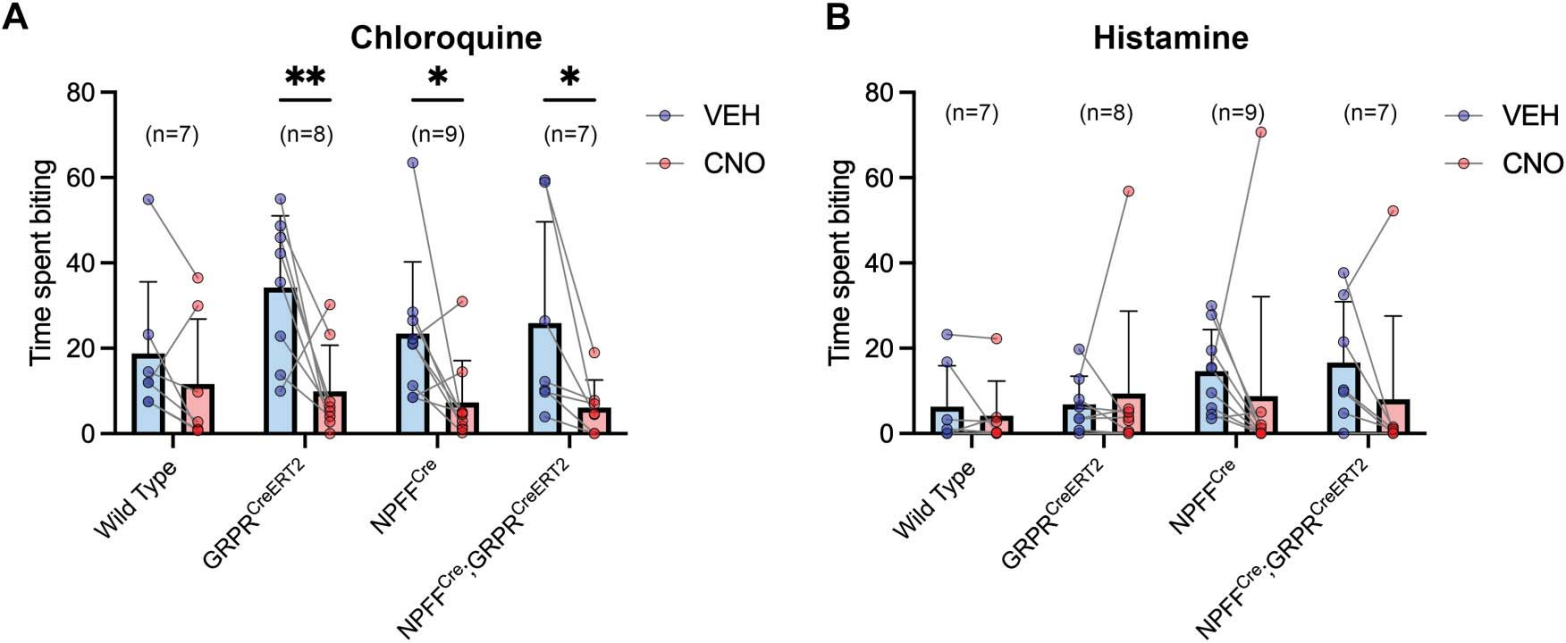
Effects of chemogenetic inhibition on itch behaviour induced by intradermal injection of chloroquine or histamine in the calf. (A) Treatment with CNO caused a significant reduction in the time spent biting the calf after injection of chloroquine in GRPR^CreERT2^, NPFF^Cre^, and GRPR^CreERT2^; NPFF^Cre^ mice, but not in wild-type animals. (B) No significant effects were observed on the biting induced by the injection of histamine. Significance: *P <0.05, **P < 0.01.

The effects of chemogenetic inhibition on pruritogen-evoked itch are illustrated in Figure 3. A 2-way ANOVA revealed that the biting evoked by intradermal injection of chloroquine into the calf was significantly reduced by treatment with CNO (*P* < 0.0001). Tukey multiple comparison tests showed that this effect of CNO was present in the GRPR^CreERT2^ (n = 8, 9.90 ± 10.80 seconds CNO vs 34.24 ± 16.83 seconds vehicle, *P* = 0.002), NPFF^Cre^ (n = 9, 7.30 ± 9.82 seconds CNO vs 23.46 ± 16.75 seconds vehicle, *P* = 0.021), and GRPR^CreERT2^; NPFF^Cre^ (n = 7, 6.13 ± 6.44 seconds CNO vs 25.87 ± 23.77 seconds vehicle, *P* = 0.014) mice (Fig. 3A). There was no significant effect of CNO treatment in the wild-type mice (n = 7, 11.63 ± 15.20 seconds CNO vs 18.79 ± 16.78 seconds vehicle, *P* = 0.347). Histamine-evoked biting was not significantly affected by treatment with CNO in any of the genotypes (Fig. 3B, effect of CNO *P* = 0.306, effect of genotype *P* = 0.647, 2-way ANOVA) (GRPR^CreERT2^, n = 8, 9.36 ± 19.31 seconds CNO vs 6.81 ± 6.65 seconds vehicle; NPFF^Cre^, n = 9, 8.78 ± 23.29 seconds CNO vs 14.61 ± 9.75 seconds vehicle; GRPR^CreERT2^; NPFF^Cre^, n = 7, 8.03 ± 19.50 seconds CNO vs 16.63 ± 14.30 seconds vehicle; wild type, n = 7, 4.18 ± 8.11 seconds CNO vs 6.32 ± 9.60 seconds vehicle).

### 3.3. The effect of chemogenetic inhibition on mechanical sensitivity before and after spared nerve injury

**Figure 4.**
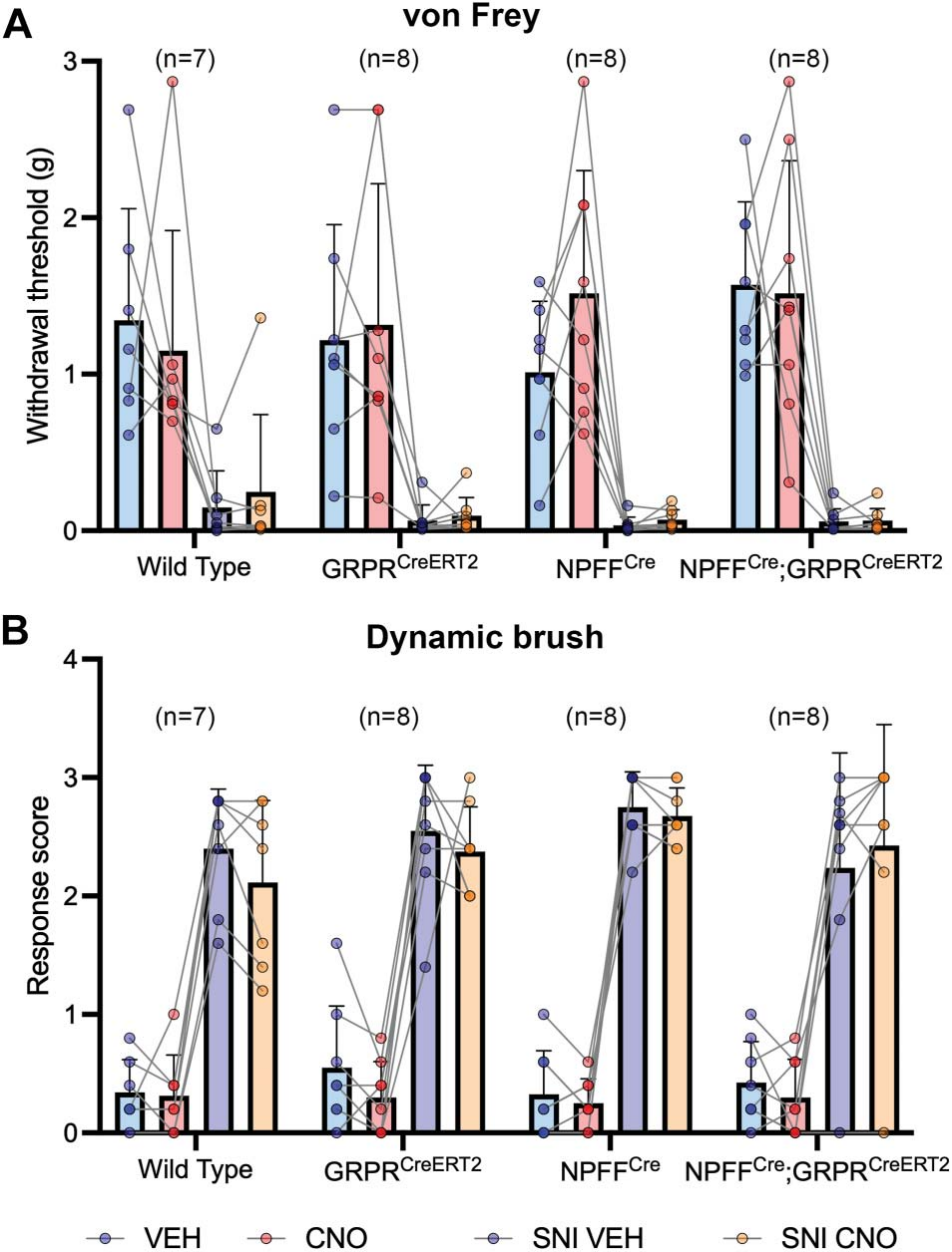
Effects of chemogenetic inhibition on responses to mechanical stimuli. (A) Withdrawal thresholds for von Frey tests. (B) Response scores for the dynamic brush test. In both cases, tests were carried out before (“VEH,” “CNO”) and after (“SNI-VEH,” “SNI-CNO”) spared nerve injury. In all groups, SNI significantly decreased von Frey withdrawal thresholds and increased dynamic brush response scores (*P* < 0.0001 in both cases). However, there were no significant differences between vehicle and CNO treatment either before SNI or after SNI.

We tested the effect of chemogenetic inhibition on the von Frey and dynamic brush tests both before and 2 to 4 weeks after SNI. Mice showed a significant reduction in threshold in the von Frey tests (*P* < 0.0001, 3-way ANOVA), and a significant increase in scores obtained in the dynamic brush tests (*P* < 0.0001, 3-way ANOVA) following SNI. However, there was no effect of treatment with CNO (von Frey, *P* = 0.468; dynamic brush, *P* = 0.120, 3-way ANOVA) and the response did not vary by genotype (von Frey, *P* = 0.773; dynamic brush, *P* = 0.671, 3-way ANOVA). Tukey multiple comparison tests demonstrated that CNO did not alter the response in any of the genotypes to von Frey or dynamic brush tests, in either the pre-SNI tests or in the tests carried out after SNI (Fig. 4).

### 3.4. The effect of chemogenetic inhibition on spontaneous neuropathic pain

**Figure 5.**
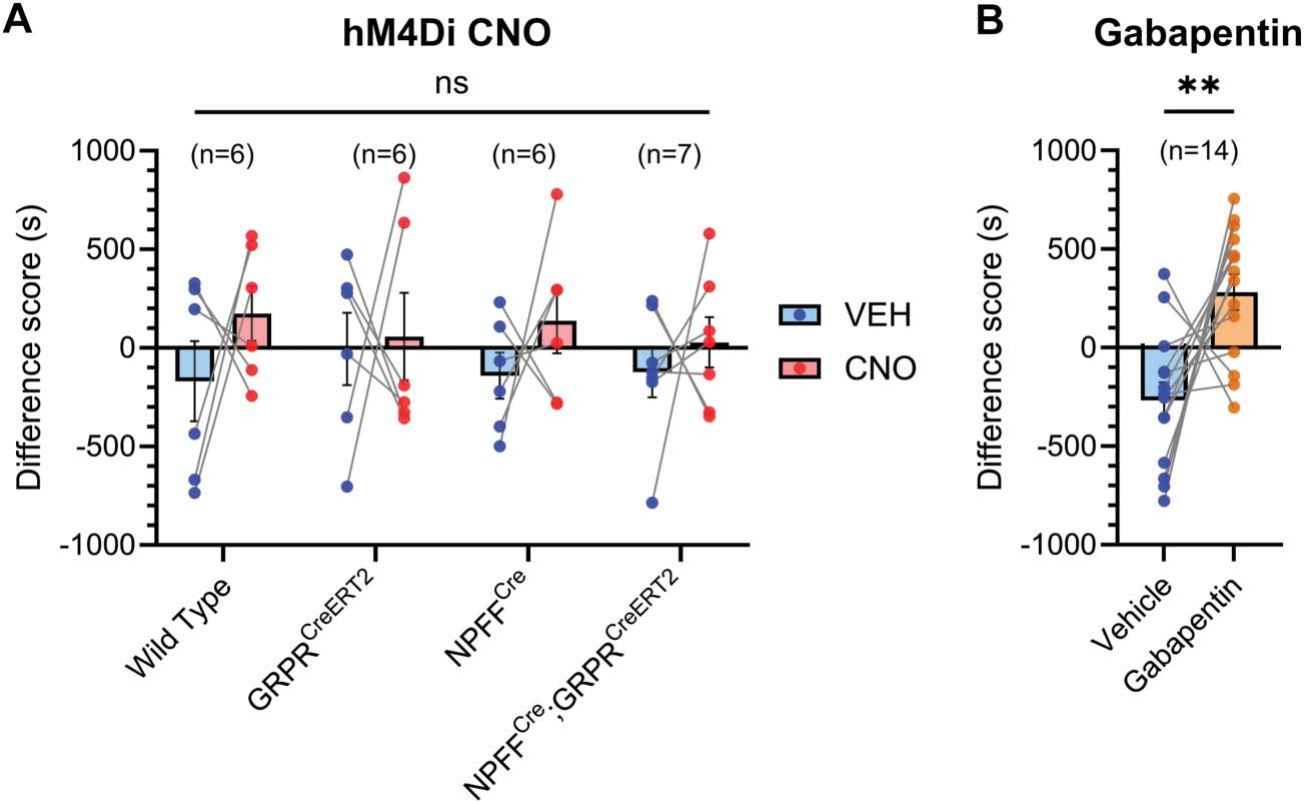
Effects of chemogenetic inhibition on spontaneous neuropathic pain. (A) Difference scores testing the ability of CNO to induce CPP. No significant differences were observed between groups. (B) Difference scores for CPP testing the ability of gabapentin to attenuate spontaneous neuropathic pain, included alongside CNO testing as a positive control. Significance: **P < 0.01.

To determine the contribution of GRPR- and NPFF-expressing dorsal horn neurons to ongoing neuropathic pain, we then tested the ability of CNO to induce conditioned place preference after SNI. No significant difference between genotypes was observed, and CNO did not induce preference or aversion in any experimental group (Fig. 5A; 2-way RM ANOVA; Genotype × Chamber interaction F_3,21_ = 0.16, *P* = 0.923; n = 6-7). In parallel experiments, included within the same experimental cohorts as CNO experiments as a positive control, we observed that gabapentin was able to induce CPP after SNI (Fig. 5B; paired *t* test; *P* = 0.0076, n = 14), demonstrating the effectiveness of the CPP protocol in detecting analgesia in the context of neuropathic pain. The lack of effect of CNO suggests that chemogenetic inhibition of GRPR- and/or NPPF-expressing dorsal horn neurons does not have a major effect in attenuating ongoing neuropathic pain, although given our sample size we cannot rule out the possibility of a more subtle contribution.

### 3.5. The effect of clozapine-N-oxide on the electrophysiological properties of vertical cells

**Figure 6.**
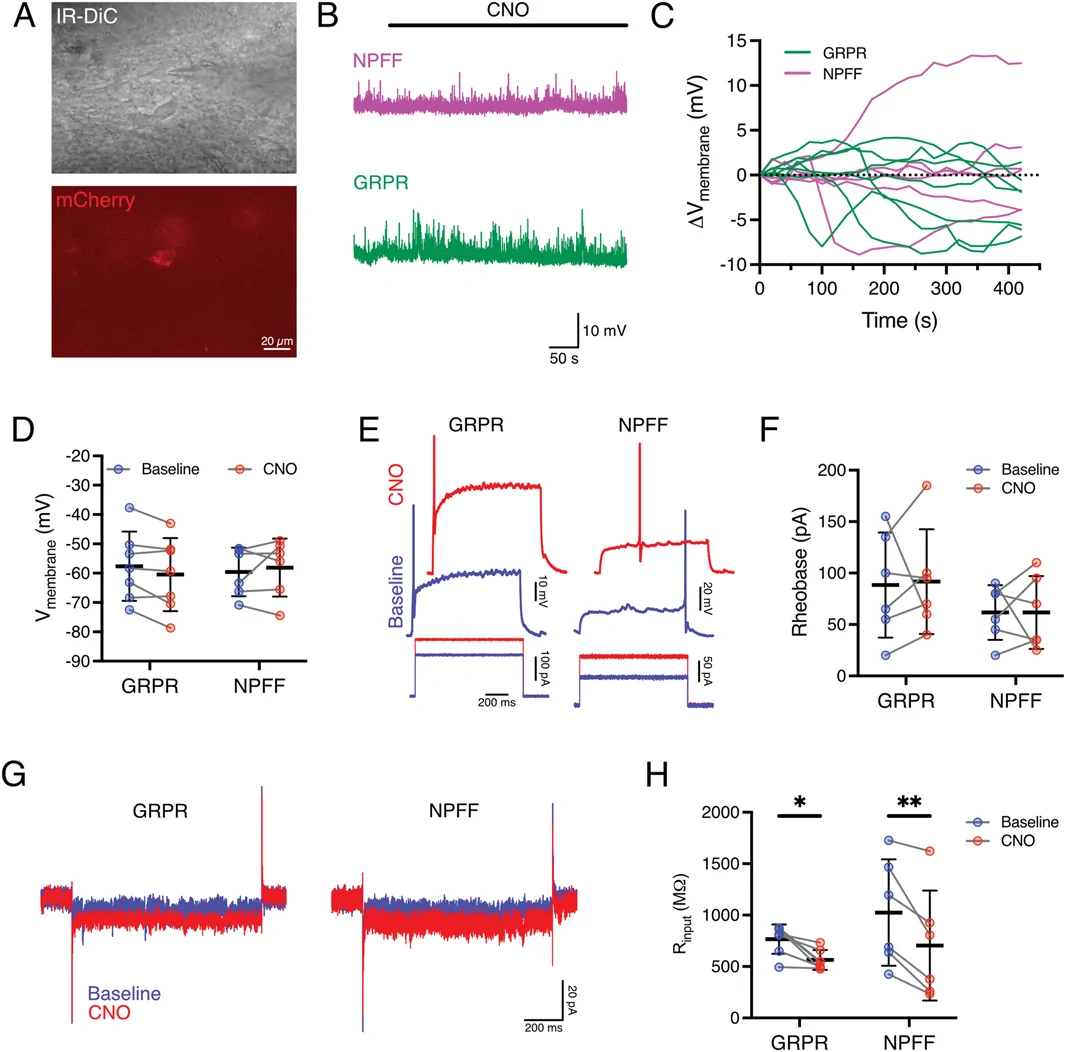
Effects of chemogenetic inhibition of the electrophysiological properties of GRPR- and NPFF-expressing cells. (A) Images of a patched NPFF cell (top) that is labelled with hM4Di-mCherry (bottom). (B) Example membrane voltage traces from NPFF and GRPR cells, recorded during the application of CNO. (C) Quantification of the membrane voltage change compared with baseline in individual GRPR and NPFF cells during CNO administration. (D) No significant differences were observed in the membrane voltage recorded in the 1 minute before (“baseline”), compared with the final minute (“CNO”) of a 7-minute CNO application. (E) Example traces showing the change in rheobase in GRPR and NPFF cells prior to (“baseline,” blue traces) and during CNO application (“CNO,” red traces). (F) Overall, CNO did not significantly alter the rheobase in either cell type. (G) Example current traces that resulted from the voltage step used to calculate input resistance, recorded before (“baseline,” blue traces) and during CNO application (“CNO,” red traces). (H) CNO significantly reduced the input resistance of both GRPR and NPFF cells. Significance: *P < 0.05, **P < 0.01.

To determine the effect of CNO on the electrophysiological properties of GRPR- and NPFF-expressing dorsal horn neurons, we performed whole-cell patch-clamp recordings from hM4Di-mCherry–labelled cells in *ex vivo* spinal cord slices (Fig. 6A). Application of CNO resulted in a hyperpolarisation of membrane potential (greater than −5 mV) in only 3/7 GRPR and 1/6 NPFF cells (Figs. 6B and C) and when the first minute (baseline) was compared with the final minute of CNO application, this demonstrated that CNO did not alter the membrane potential of either cell type (Fig. 6D; GRPR; n = 7, −57.6 ± 11.8 vs −60.5 ± 12.5 mV, NPFF; n = 6, −59.6 ± 8.3 vs −58.1 ± 9.9 mV; Genotype effect *P* = 0.970, CNO effect *P* = 0.629, 2-way RM ANOVA). Application of CNO increased the rheobase in 4/6 GRPR and 3/6 NPFF cells, but overall, this was not significantly different (Fig. 6E and F; GRPR; n = 6, 88.3 ± 51.2 vs 91.7 ± 50.9 pA, NPFF; n = 6, 61.7 ± 25.6 vs 61.7± 35.4 pA; Genotype effect *P* = 0.200, CNO effect *P* = 0.901, 2-way RM ANOVA). All cells tested, except for 1 GRPR cell, exhibited a reduction in input resistance following CNO application, and this response did not differ by cell type (Fig. 6G and H; GRPR; n = 7, 766.5 ± 142.9 vs 563.7 ± 96.7 MΩ, NPFF; n = 6, 1024.3 ± 517.6 vs 704.1± 534.5 MΩ; Genotype effect *P* = 0.337, CNO effect *P* = 0.0001, 2-way RM ANOVA).

Although expression levels varied between cells, we consistently patched neurons that had the brightest mCherry fluorescence. It is therefore unlikely that the failure to detect consistent changes in membrane potential in the presence of CNO was due to low expression of hM4Di, although we cannot rule out this possibility.

## 4. Discussion

Our main findings are that although chemogenetic inhibition of GRPR and/or NPFF cells suppressed chloroquine-evoked itch behaviour, it had no detectable effect on baseline responses to mechanical stimuli, on mechanical hypersensitivity after SNI, or on SNI-evoked ongoing pain as measured by CPP.

### 4.1. A role for gastrin-releasing peptide receptor and neuropeptide FF cells in itch

There is considerable evidence that GRPR-expressing excitatory interneurons are involved in itch mechanisms. Ablation of GRPR neurons with intrathecal bombesin-saporin reduced pruritogen-evoked itch behaviours but had no effect on responses to painful stimuli.^51^ This can be attributed to GRPR itself, as mice lacking the receptor showed reduced pruritogen-evoked itch, while intrathecal GRPR antagonists reduced itch-like behaviours in response to pruritogens in wild-type, but not GRPR^−/−^ mice.^50^ Mu et al.^34^ demonstrated that most GRPR-expressing spinal neurons are interneurons and that these interneurons are directly linked to spinoparabrachial neurons through glutamatergic synapses. They also showed that optogenetic inhibition of these projection neurons suppresses itch-related behaviours. Although it was originally suggested that GRP was released from primary afferent neurons,^50^ it is now thought that the peptide is not normally present in these cells, but is expressed by excitatory interneurons in the SDH.^13,17,19,22,47,56^ Pagani et al.^38^ further demonstrated that GRP-containing spinal interneurons formed synapses with the GRPR-expressing interneurons, and that co-release of glutamate and GRP was required to activate the GRPR cells and evoke itch.

Based on these findings, it is not surprising that chemogenetic inhibition of GRPR neurons suppressed itch evoked by chloroquine. However, we did not observe any significant change in the responses to histamine. This may be because GRP-GRPR signalling in the spinal cord is more important for nonhistaminergic itch, compared with that evoked by histamine.^2,8^ In addition, we have found that the biting behaviour evoked by intradermal injection of histamine is less consistent than that observed with chloroquine, making it more difficult to demonstrate a significant suppression.

Less is known about the function of SDH interneurons that express NPFF, although we showed that some of them respond to pruritic and noxious stimuli.^21^ We had previously examined the relationship between these 2 populations of vertical cells by generating GRPR^Flp^; NPFF^Cre^ mice, and injecting AAVs coding for Cre- and Flp-dependent fluorescent proteins.^45^ We found that while neurons containing the precursor protein pro-NPFF showed no overlap with GRPR-expressing cells, there was considerable overlap between Cre- and Flp-positive cells that lacked pro-NPFF. This suggests that some GRPR-expressing neurons may have been captured in the NPFF^Cre^ mouse, and silencing of these may have contributed to the anti-pruritic effect observed in chemogenetic experiments involving this line. It is also likely that cells expressing NPFF, but not GRPR, are involved in mediating itch, as around one-third of pro-NPFF-immunoreactive neurons in SDH were activated by intradermal chloroquine.^21^ In addition, NPFF-Cre cells that lack GRPR and pro-NPFF may contribute to itch responses.

Interestingly, there was little difference in the degree of itch suppression among the 3 genotypes tested, raising the possibility that cells that express both GRPR and NPFF are particularly important for itch.

### 4.2. Potential mechanisms of action of hM4Di

The inhibitory DREADD receptor hM4Di acts on various biochemical pathways to suppress both the excitability of neurons and synaptic transmission. These inhibitory mechanisms include direct effects at the soma/dendritic level, such as suppression of Na^+^ and HCN channels, and opening of GIRK K^+^ channels, leading to membrane hyperpolarisation and thus a direct reduction of neuronal excitability. This would be detected as a hyperpolarisation and an increase in rheobase in current-clamp recordings. However, apart from a decrease in input resistance, presumably reflecting channel opening, changes in membrane potential and rheobase were inconsistent in both GRPR and NPFF populations.

An additional mechanism involves inhibiting voltage-gated Ca^2+^ channels in axon terminals, thereby reducing neurotransmitter release.^49^ This would be independent of any effects on the cell body but would require targeting of the hM4Di receptor to axon terminals. As the construct used in this study consisted of hM4Di fused to mCherry, our finding of numerous mCherry-labelled axonal boutons confirms that the receptor was indeed expressed at these sites. Because of the relatively modest and inconsistent effects observed during CNO application in electrophysiological recordings, it is likely that this presynaptic action, reducing transmitter release from the GRPR and NPFF cells, was the main mechanism underlying the suppression of chloroquine-induced itch.

### 4.3. Lack of evidence for a role for gastrin-releasing peptide receptor and neuropeptide FF cells in neuropathic mechanical allodynia

As noted above, vertical cells have been implicated in mechanical allodynia/hyperalgesia based on their morphological properties: their dendrites often lie within the A-LTMR receiving zone, which extends ventrally from lamina IIi,^20^ and their axons can innervate lamina I projection neurons.^11,27^ There is some anatomical evidence that ventral dendrites of vertical cells receive direct synaptic input from A-LTMRs,^3,46,61^ but a more complex polysynaptic circuit linking these cell types, and involving 3 different classes of excitatory interneuron was suggested by Lu et al.^26^ They proposed that A-LTMRs innervated PKCγ-expressing neurons, which were presynaptic to a class known as “transient central” cells,^20^ and that these in turn synapsed onto vertical cells. A feedforward glycinergic inhibitory mechanism operating at the level of the PKCγ cell was believed to prevent the circuit opening under normal conditions.^26^ We had found that chemogenetic activation of GRPR-expressing cells in the L3-5 segments caused biting of the affected skin region (presumably reflecting itch), as well as pain-like behaviours, including licking, lifting/guarding, and shaking of the paw, together with reduced von Frey thresholds.^42^ We had therefore expected that inhibiting these cells would suppress the mechanical hypersensitivity observed after SNI, and possibly also reduce ongoing pain. However, we saw no reduction in behavioural signs associated with neuropathic pain. Although chemogenetic activation can provide insights into neuronal function and circuitry, the simultaneous activation of neuronal populations is clearly unphysiological, making the interpretation of behavioural results difficult. By contrast, chemogenetic inhibition tests whether the neurons in question are required for a particular behavioural response. Taken together with our previous findings,^42^ the present results suggest that GRPR and NPFF cells play an essential role in mediating itch but may not contribute to neuropathic pain. However, we cannot rule out the possibility of distinct “pruritic” and “nociceptive” subsets among these cells, with the former being more susceptible to chemogenetic inhibition. Interestingly, cells that express Cre under control of the GRP promoter in a BAC transgenic line (GRP::Cre) often show transient firing in response to injected current, and may therefore correspond to the transient central cells.^13,38^ This suggests that the synapses linking the transient central to vertical cells identified by Lu et al.^26^ may, in fact, be between GRP- and GRPR-expressing neurons^38^ and function as part of an itch circuit, rather than being in a pathway for mechanical allodynia.

During the course of this study, Noh et al.^37^ posted a preprint reporting that chemogenetic inhibition of GRPR cells caused a dramatic reduction in mechanical hypersensitivity in both neuropathic and inflammatory models. Specifically, it reversed the 50% paw withdrawal threshold (measured with von Frey hairs) and mechanical hypersensitivity (tested with cotton swabs) in 2 different diabetic models, as well as following SNI, and in the complete Freund adjuvant (CFA) inflammatory model. Since our findings for mechanical hypersensitivity following SNI are very different from those of Noh et al,^37^ it is important to consider whether this could be explained by differences in the experimental technique. They used a different GRPR^Cre^ line, but validated it by showing that >90% of the neurons targeted by intraspinal injection of AAV8.hSyn.Cre^ON^.hM4Di-mCherry contained *Grpr* message. We have demonstrated a high degree of fidelity in GRPR cells from the GRPR^CreERT2^ mouse line that we used, with 88% of Cre+ cells having at least 4 *Grpr* transcripts (and 99% having at least one).^42^ Both our study and that of Noh et al. used the same viral vector to deliver hM4Di, and this was obtained from the same supplier (Addgene). However, the amount that Noh et al. delivered was considerably greater, equivalent to 2.5 × 10^9^ gene copies given as a single injection,^37^ whereas we delivered 2.2 × 10^8^ gene copies at each of 3 injection sites. This means that the total number of viral particles delivered by Noh et al. was ∼3.8-fold greater than in our study. Noh et al. made their injections through the T12-T13 intervertebral space, which they describe as targeting the L3-L4 spinal segments, and although it is not clear how far along the spinal cord the viral transfection spread, there will presumably have been much higher expression at the level of their injection site than would have occurred in our study. It is therefore possible that relatively low levels of hM4Di-mediated inhibition of GRPR cells are sufficient to suppress itch, whereas much higher levels of inhibition are needed to reverse mechanical allodynia. One way to test this (while avoiding high levels of hM4Di receptor expression) would be to use chemogenetic^30^ or optogenetic^31^ methods that operate through Cl^−^ channels to produce a shunting inhibition, as this should provide a much more potent silencing of these neuronal populations.^60^

## Conflict of interest statement

The authors have no conflict of interest to declare.
